# Supplementary material for: Criteria-based curation of a therapy-focused compendium to support treatment recommendations in precision oncology
Source: NPJ Precis Oncol. 2021 Jun 23;5:58. doi: 10.1038/s41698-021-00194-z (PMC8222322; doi:10.1038/s41698-021-00194-z)
Supplement: Supplementary file 1 — Supplementary Information [file 41698_2021_194_MOESM1_ESM.pdf]

Criteria-based curation of a therapy-focused compendium to support treatment recommendations in precision oncology

Frank P. Lin [1,2,3,*], Subotheni Thavaneswaran [2,3,4], John P. Grady [3,4], Mandy Ballinger [3,4], Maya Kansara [3,4], Samantha R. Oakes [3,4], Jayesh Desai [5], Chee Khoon Lee [2,6], John Simes [2], David M. Thomas [3,4,*]

1. Kinghorn Centre for Clinical Genomics, Garvan Institute of Medical Research, Sydney, NSW, Australia
2. NHMRC Clinical Trial Centre, University of Sydney, Sydney, NSW, Australia
3. St Vincent's Clinical School, Faculty of Medicine, UNSW Sydney, NSW, Australia
4. The Kinghorn Cancer Centre, Garvan Institute of Medical Research, Sydney, NSW, Australia
5. Department of Medical Oncology, Peter MacCallum Cancer Centre, Melbourne, VIC, Australia
6. Department of Medical Oncology, St George Hospital, Kogarah, NSW, Australia

Supplementary Table 1: An excerpt from TOPOGRAPH showing differential tiering of tumour mutational burden as a predictive biomarker for immune checkpoint inhibitors.

Tier	Biomarker	Alteration	Cancer type	Therapy	Comments
2	TMB	High	Cervical cancer<br>Endometrial cancer<br>Neuroendocrine tumour<br>Salivary gland carcinoma<br>Small-cell lung cancer<br>Thyroid cancer<br>Vulvar cancer	Pembrolizumab	Not TGA approved. FDA approved. In Phase 2 KEYNOTE-158 trial, the following cancer types have higher response rate in TMB high group compared to non-TMB-H groups.
2	TMB	High	Non-small cell lung cancer	Nivolumab + Ipilimumab	Not TGA approved. Phase 3 CHECKMATE 227. Prespecified, prospective TMB cutoff of 10mut/MB in 57% of cohort. Median PFS: 7.2 vs 5.5 months. Quantitative TMB is not correlated with TMB.
3	TMB	High	Non-small cell lung cancer	Durvalumab + Tremelimumab	Pre-planned exploratory biomarker analysis from the Phase 3 MYSTIC trial.
3	TMB	High	Solid tumours except Colorectal Cancer	Pembrolizumab	Overall Tier 3 based on phase 2 KEYNOTE-158 trial, forming the basis of FDA pan-cancer approval noted. In overall cohort, TMB-H (v non-TMB-H) was noted to have an objective response rate of 29% (versus 6%).
4	TMB	High	Colorectal Cancer	Pembrolizumab	Microsatellite-stable colorectal cancers were not included/reported in KEYNOTE-158.
4	TMB	High	Non-small cell lung cancer	Nivolumab	Exploratory analysis from Checkmate 026.
4	TMB	High	Gastric cancer	Toripalimab	Exploratory analysis showed ORR 33% v 6% at TMB threshold of 12/MB

Abbreviations TMB: Tumour Mutational Burden.

Supplementary Table 2. Summary of biomarkers and therapies curated in TOPOGRAPH by tiers

Item	Total	T1-4	Tier 1A		Tier 1B		Tier 2		Tier 3		Tier 4		Tier 1/2		Tier 3/4		Tier R1		Tier R2	
	N	N	N	(%)[*]	N	(%)	N	(%)	N	(%)	N	(%)	N	(%)	N	(%)	N	(%)[†]	N	(%)
**1. Biomarkers**																				
**Number of biomarkers**	199	178	24	(13)	33	(19)	43	(24)	49	(28)	146	(82)	61	(34)	158	(89)	9	(5)	76	(38)
**Number of unique biomarkers by type of alterations**																				
Simple mutations	463	284	10	(4)	8	(3)	93	(33)	91	(32)	143	(50)	100	(35)	211	(74)	30	(6)	268	(58)
Variant groups	181	156	4	(3)	6	(4)	19	(12)	38	(24)	127	(81)	22	(14)	149	(96)	3	(2)	34	(19)
Protein expressions	68	59	9	(15)	12	(20)	12	(20)	9	(15)	40	(68)	21	(36)	44	(75)	-		11	(16)
Amplification/deletions	42	33	1	(3)	1	(3)	3	(9)	3	(9)	31	(94)	3	(9)	32	(97)	-		24	(57)
Fusion/rearrangements	48	43	5	(12)	10	(23)	17	(40)	9	(21)	22	(51)	25	(58)	26	(60)	-		8	(17)
Splice variants	2	2	-		-		-		-		2	(100)	-		2	(100)	-		1	(50)
Gene methylations	5	5	1	(20)	-		-		-		4	(80)	1	(20)	4	(80)	-		-	
Germline mutations	9	9	4	(44)	2	(22)	3	(33)	5	(56)	3	(33)	5	(56)	7	(78)	-		2	(22)
Combination markers	63	45	2	(4)	2	(4)	3	(7)	9	(20)	30	(67)	6	(13)	39	(87)	-		22	(35)
Complex biomarkers	6	6	-		3	(50)	5	(83)	2	(33)	4	(67)	6	(100)	4	(67)	-		-	
**All alterations**	887	642	36	(6)	44	(7)	155	(24)	166	(26)	406	(63)	189	(29)	518	(81)	33	(4)	370	(42)
**2. Cancer types**																				
**Unique cancer types**	106	105	30	(29)	26	(25)	46	(44)	46	(44)	56	(53)	64	(61)	78	(74)	6	(6)	32	(30)
Common	17	17	8	(47)	7	(41)	10	(59)	11	(65)	15	(88)	11	(65)	16	(94)	2	(12)	12	(71)
Less common	16	16	5	(31)	4	(25)	9	(56)	12	(75)	9	(56)	10	(62)	15	(94)	-		3	(19)
Rare	73	72	17	(24)	15	(21)	27	(37)	23	(32)	32	(44)	43	(60)	47	(65)	4	(5)	17	(23)
**3. Therapies**																				
**Number of therapies**	373	345	55	(16)	37	(11)	65	(19)	92	(27)	219	(63)	130	(38)	271	(79)	15	(4)	118	(32)
**Number of unique therapies by cancer type**																				
Histotype agnostic	130	117	1	(1)	3	(3)	-		3	(3)	114	(97)	4	(3)	115	(98)	2	(2)	33	(25)
Breast cancer	83	72	16	(22)	11	(15)	11	(15)	13	(18)	30	(42)	38	(53)	41	(57)	-		29	(35)
Lung cancer (NSCLC)	82	72	9	(12)	4	(6)	11	(15)	33	(46)	45	(62)	19	(26)	65	(90)	5	(6)	32	(39)
Colorectal cancer	30	24	3	(12)	1	(4)	4	(17)	9	(37)	11	(46)	8	(33)	18	(75)	3	(10)	13	(43)
Gastric/GOJ cancers	22	17	2	(12)	-		3	(18)	4	(24)	9	(53)	5	(29)	12	(71)	-		6	(27)
Glioma, meningioma	21	18	2	(11)	-		2	(11)	2	(11)	14	(78)	3	(17)	16	(89)	-		3	(14)
Ovarian and peritoneal	21	21	1	(5)	2	(10)	4	(19)	2	(10)	18	(86)	5	(24)	19	(90)	-		-	
Urothelial cancer	19	18	1	(6)	2	(11)	3	(17)	2	(11)	11	(61)	6	(33)	13	(72)	-		2	(11)
Prostate cancer	18	9	-		-		3	(33)	1	(11)	7	(78)	3	(33)	8	(89)	-		11	(61)
Cutaneous Melanoma	16	14	4	(29)	1	(7)	2	(14)	4	(29)	5	(36)	7	(50)	8	(57)	-		6	(37)
Pancreatic cancer	14	13	2	(15)	-		2	(15)	1	(8)	8	(62)	4	(31)	9	(69)	-		2	(14)
Sarcomas	14	12	-		1	(8)	2	(17)	2	(17)	9	(75)	3	(25)	10	(83)	-		2	(14)
Endometrial & uterine	13	13	1	(8)	1	(8)	1	(8)	6	(46)	4	(31)	3	(23)	10	(77)	-		-	
Acute lymphoblastic leukaemia	11	11	5	(45)	-		6	(55)	1	(9)	3	(27)	8	(73)	4	(36)	4	(36)	-	
Gastrointestinal stromal tumours	11	10	3	(30)	1	(10)	6	(60)	2	(20)	2	(20)	7	(70)	4	(40)	2	(18)	7	(64)
Biliary tract cancers	10	9	-		-		2	(22)	4	(44)	3	(33)	2	(22)	7	(78)	-		3	(30)
Chronic Mylogenous leukaemoa	6	6	4	(67)	1	(17)	5	(83)	1	(17)	-		5	(83)	1	(17)	4	(67)	1	(17)
Other haematological	36	32	7	(22)	12	(37)	8	(25)	5	(16)	4	(12)	26	(81)	9	(28)	-		8	(22)
Other solid tumours	68	63	5	(8)	5	(8)	10	(16)	15	(24)	36	(57)	17	(27)	48	(76)	-		12	(18)
**Therapies-cancer type pairs**	625	551	66	(12)	45	(8)	85	(15)	110	(20)	333	(60)	173	(31)	417	(76)	20	(3)	170	(27)
**All biomarker alteration-cancer types**	1329	985	57	(6)	66	(7)	255	(26)	209	(21)	545	(55)	328	(33)	718	(73)	47	(4)	453	(34)
**Unique triplets**	2810	1754	109	(6)	86	(5)	389	(22)	303	(17)	884	(50)	577	(33)	1182	(67)	95	(3)	968	(34)

From A total of 345 therapies received Tiers 1-4 designation, 130 therapies (38%, including both drug and drug combinations) had standard-of-care indications (T1/T2), whereas 271 (79%) therapies were designated investigational tiers (T3/T4). For 56 standard-of-care therapies (of 130, 43%), there are also curated entries in T3/4 categories. NB: (*) percentages listed in Tiers 1, 1B, 2, 3, 4 have denominator of a total of Tiers 1-4 therapies (†) percentages listed in Tiers R1 and R2 references to denominator that includes all entries (include T1-4, R1/2). Percentage may not add up to 100% as a biomarker, cancer type, or therapy may appear in more than one tier. Abbreviation: GOJ: gastroesophageal junction; NSCLC: non-small cell lung cancer.

Supplementary Table 3. Comparison of curated contents in TOPOGRAPH with other public knowledge bases with respect to *ERBB2* A775_G776insYVMA in non-small cell lung cancer.

Knowledgebase	Focus of curation, Tiering / LOE scale	Curated therapies (including combinations)		Therapies where the index mutation is associated with the highest tier/LOE	
		N	List	Sensitising	Resistant
TOPOGRAPH	Therapy	6	AFA, DAC, NER, POZ, PYR, T-DM1	T-DM1 (3)<br>PYR (3)<br>POZ (3)	AFA (R2)<br>DAC (R2)<br>NER (R2)
CIViC	Variant	4	AFA, DAC, Sirolimus, T-DM1	T-DM1(Level C)<br>AFA (Level C)	DAC (Level B)
CGI	Variant	2	NER, T-DM1	T-DM1, NER	None
JAX-CKB	Not assessable [1]	13	AFA, DAC, ERL, GEF, NER, POZ, PYR, T-DM1, T-DM1 + POZ, TRA +/- chemotherapy	DAC (“Phase II”)<br>POZ (“Phase II”)<br>PYR (“Phase II”)	AFA, DAC, ERL, NER, GEF
OncoKB	Variant	2	Neratinib, T-DM1	T-DM1 (LOE 2)	None

All databases were accessed on 08 March 2021. Notes: 1) JAX-CKB reported a “25-tier ranking system” but not presented in the associated publication. The assumption made using highest tier. Abbreviations: AFA: Afatinib; DAC: Dacomitinib; ERL: Erlotinib; GEF: Gefitinib; LOE: level of evidence; NER: Neratinib; POZ: Poziotinib; PYR: Pyrotinib; TRA: Trastuzumab; T-DM1: Ado-Trastuzumab Emtansine.
